# Influence of low tumor content on tumor mutational burden estimation by whole‐exome sequencing and targeted panel sequencing

**DOI:** 10.1002/ctm2.415

**Published:** 2021-05-06

**Authors:** Wenxin Zhang, Ruixia Wang, Huan Fang, Xiangyuan Ma, Dan Li, Tao Liu, Zhenxi Chen, Ke Wang, Shiguang Hao, Zicheng Yu, Zhili Chang, Chenglong Na, Yin Wang, Jian Bai, Yanyan Zhang, Fang Chen, Miao Li, Chao Chen, Liangshen Wei, Jinghua Li, Xiaoyan Chang, Shoufang Qu, Ling Yang, Jie Huang

**Affiliations:** ^1^ Department of In Vitro Diagnostic Reagent National Institutes for Food And Drug Control (NIFDC) Beijing China; ^2^ Department of In Vitro Diagnostic Reagent Beijing Institute of Medical Device Testing Beijing China; ^3^ Geneplus‐Beijing Beijing China; ^4^ Nanjing Geneseeq Technology Inc. Nanjing China; ^5^ Berry Oncology Corporation Beijing China; ^6^ MGI, BGI‐Shenzhen Shenzhen China; ^7^ YuceBio Technology Co., Ltd. Shenzhen China; ^8^ GeneWell Biotech Ltd. Shenzhen China; ^9^ Department of Pathology, Peking Union Medical College Hospital, Chinese Academy of Medical Sciences and Peking Union Medical College Tsinghua University Beijing China; ^10^ Geneplus‐Suzhou Biomedical Engineering Corporation Suzhou China

**Keywords:** biomarker, targeted panel sequencing, tumor mutational burden, whole‐exome sequencing

## Abstract

**Background:**

Tumor mutational burden (TMB) is a promising biomarker for stratifying patient subpopulation who would benefit from immune checkpoint blockade (ICB) therapies. Although great efforts have been made for standardizing TMB measurement, mutation calling and TMB quantification can be challenging in samples with low tumor content including liquid biopsies. The effect of varying tumor content on TMB estimation by different assay methods has never been systematically investigated.

**Method:**

We established a series of reference standard DNA samples derived from 11 pairs of tumor–normal matched human cell lines across different cancer types. Each tumor cell line was mixed with its matched normal at 0% (control), 1%, 2%, 5%, and 10% mass‐to‐mass ratio to mimic the clinical samples with low tumor content. TMB of these reference standards was evaluated by both ∼1000× whole‐exome sequencing (wesTMB) and targeted panel sequencing (psTMB) at four different vendors. Both regression and classification analyses of TMB were performed for theoretical investigation and clinical practice purposes.

**Results:**

Linear regression model was established that demonstrated in silico psTMB determined by regions of interest (ROI) as a great representative of wesTMB based on TCGA dataset. It was also true in our reference standard samples as the predicted psTMB interval based on the observed wesTMB captured the intended 90% of the in silico psTMB values. Although ∼1000× deep WES was applied, reference standard samples with less than 5% of tumor proportions are below the assay limit of detection (LoD) of wesTMB quantification. However, predicted wesTMB based on observed psTMB accurately classify (>0.97 AUC) for TMB high and low patient stratification even in samples with 2% of tumor content, which is more clinically relevant, as TMB determination should be a qualitative assay for TMB high and low patient classification. One targeted panel sequencing vendor using an optimized blood psTMB pipeline can further classify TMB status accurately (>0.82 AUC) in samples with only 1% of tumor content.

**Conclusions:**

We developed a linear model to establish the quantitative correlation between wesTMB and psTMB. A set of DNA reference standards was produced in aid to standardize TMB measurements in samples with low tumor content across different targeted sequencing panels. This study is a significant contribution aiming to harmonize TMB estimation and extend its future application in clinical samples with low tumor content including liquid biopsy.

## Graphical abstract

Established 11 sets of reference standard samples with variable tumor proportions for evaluating TMB estimation.

In silico and experimentally verified the linear regression model between the TMB analyzed by deep whole‐exome sequencing and targeted panel sequencing in reference standard samples.

Targeted panel sequencing method might outperform WES for TMB estimation and categorization in samples with low tumor proportion.

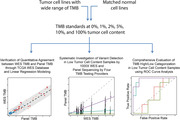

## 1 INTRODUCTION

Tumor mutational burden (TMB) is a promising cancer biomarker for predicting and monitoring immune checkpoint blockade (ICB) therapies’ efficacy.^1^, ^2^, ^3^, ^4^, ^5^, ^6^ It is postulated that a high TMB promotes the presentation of tumor neoantigens on the plasma membrane, triggering the host immune responses.^7^, ^8^, ^9^, ^10^ Numerous studies have reported the feasibility of utilizing TMB in monitoring therapeutic outcomes of ICB in treating various cancers.^11^, ^12^, ^13^, ^14^ Several immune checkpoint inhibitors have been approved by FDA to treat various solid tumors and hematological malignancies.^15^ However, despite of the increasing popularity, quantifying TMB in clinical testing setup faces several technical challenges.^16^, ^17^ Whole‐exome sequencing (WES) is often considered as the gold standard method for TMB measurement (wesTMB). Unfortunately, the clinical utility of WES is limited by its relatively high cost, inaccessibility to tissue biopsy specimen, and poor analytical sensitivity for detecting somatic variants with low variant allelic frequency (VAF) at low sequencing coverage depth. To circumvent these difficulties, laboratories usually adopted targeted panel sequencing instead to estimate TMB (psTMB), which also has the potential to be applied on liquid biopsy samples when tumor tissue is unavailable.^18^, ^19^

Valid concerns have been raised over the comparability of psTMB to wesTMB for two reasons: (1) the region of interest (ROI) selected at panel design often focuses on a small subset of mutational hotspots or cancer‐associated genes. Limited size of ROI reduces TMB counts and introduces sampling bias if the variants are not evenly distributed across the exome.^20^, ^21^, ^22^, ^23^ (2) The coverage depth of panel‐sequencing is often deeper than WES, which increases the detection sensitivity for somatic variants with low VAF in panel sequencing compared to traditional low‐depth WES. To date, the application of psTMB with various ROIs has been carefully investigated by Friends of Cancer Research (Friends) and the Quality Assurance Initiative Pathology (QuIP).^24^, ^25^ They have demonstrated the consistency of wesTMB and psTMB from 11 vendors through in silico simulation using 10 tumor cell lines.^25^ However, all these studies used datasets generated from tumor samples with relatively high tumor content,^26^ normally above 20%. As the consequence of low tumor cell content of tumor biopsy, low VAF variants, are frequently encountered in clinical testing scenario, which significantly influences the sensitivity of mutation calling and TMB estimation and has not been explored in detail. Understanding the influence of low VAF variants on TMB estimation becomes particularly important for blood‐based TMB (bTMB) estimation in liquid biopsy, which provides a less invasive surrogate for patients whose tissue biopsy could not be obtained but generally shows much lower VAF than tissue biopsy.^27^, ^28^

In this study, we aimed to conduct a systematic investigation on TMB estimation in samples with low tumor proportions and by using different experimental approaches. We first built a linear model through in silico computation to verify the correlation between wesTMB and psTMB. We further produced and characterized 12 series of reference standard DNA samples derived from tumor–normal paired cell lines, and experimentally diluted the reference standards to imitate samples of low tumor fraction. TMB estimation within the reference samples was performed using deep WES and targeted panel sequencing from different commercial vendors. Comparative test was applied to challenge both the quantitative coherence of TMB measurement by different vendors and the validity of our in silico model. Our study contributed to reconciling TMB measurements in samples with low VAF, and developing a fundamental standardized system to assess diverse TMB estimation products, which will guide the future application of psTMB in low tumor purity tissue biopsies and liquid biopsies.

## 2 MATERIALS AND METHODS

### 2.1 In silico linear modeling of psTMB and wesTMB in TCGA data

#### 2.1.1 Sample and variant filtering based on sequencing metrics

We downloaded the MC3 data from TCGA (https://gdc.cancer.gov/about‐data/publications/mc3‐2017)^29^ and included 24 major cancer types (BLCA, BRCA, CESC, CHOL, COAD, DLBC, ESCA, GBM, HNSC, KIRC, KIRP, LGG, LIHC, LUAD, LUSC, MESO, OV, PAAD, READ, SKCM, STAD, UCEC, UCS, UVM) in this study. According to MC3 recommended sample and variant filtering guideline (https://www.synapse.org/#!Synapse:syn7214402/wiki/406007), we filtered out samples processed by whole‐genome amplification (WGA) method, nonpreferred sample pairs, or samples analyzed by sequence gap‐filler. We also excluded the samples whose maximum somatic allelic frequency (MSAF) was lower than 10%. In addition, mutations of G>T artifacts bias, common germline/artifact in ExAC v0.3 or likely oxo‐G artifact^30^ were filtered out. A sample was also excluded from the analysis if 50% or more of its mutations were filtered out by the above rules. Finally, the retained mutations must also display at least 25× depth of coverage at the variant site with at least three supporting sequencing reads, at least 5% of variant allelic frequency (VAF), less than 1% frequency within ExAC database, and less than 1% frequency within ExAC East Asian sub‐database.

#### 2.1.2 Variant filtering based on annotated effects

For calculating wesTMB, mutations encompassing small insertions and deletions (Indels), missense mutations, nonsense mutations, nonstop mutations, and mutations in splice sites were included. For psTMB estimation, mutations within ROI of the panel including 50 bp flanking regions on both ends of each region were included. Three additional well‐recognized variants retention rules for psTMB calculation were also applied for comparison. Briefly, rule 1 will retain nonsynonymous somatic SNV or INDEL within 2 bp upstream and downstream of coding sequences regardless of functional annotations.^31^ Rule 2 will remove tumor driver mutations while retain synonymous variants.^7^ Under the filtering criteria presented in this study (new rule), a different set of tumor driver mutations compared to rule 2 was implemented and removed, which better represents the driver mutations covered by the targeted panels used in this study, whereas the other variants were retained toward TMB summation. Both wesTMB and psTMB values were normalized to mutation count per million base pair of coding regions (mut/Mb).

#### 2.1.3 Linear model fitting

linear regression analysis was performed by R (version 3.6.2) function “lm” to derive the fitting formula of psTMB and wesTMB. Ninety percent prediction intervals were calculated by R function “predict” to estimate fitting variance. Three variants retention rules for psTMB calculation were compared by Pearson, Spearman correlation, and *R* ^2^ analyses. Finally, we chose the optimal fitting relationship computed by new rule for further analysis.

### 2.2 Preparation of low tumor content standard samples for TMB measurement

A total of 24 stable cell lines, derived from 11 tumor–normal pairs (TMB‐1, ‐2, ‐4, ‐5, ‐6, ‐7, ‐8, ‐9, ‐11, ‐12, ‐13) and one pair of healthy donors (TMB‐14) (Table S1), were included for this study. Review and approval by ethics committee is not applicable for these stable cell lines. The identity of each cell line was confirmed by STR genotyping, and cultured cells were harvested for genomic DNA extraction. WES was performed on each parental tumor cell line by hybridization capture using IDT xGen Exome Research Panel v1.0 and Illumina Hiseq X Ten platform with PE150 chemistry for raw TMB estimation according to the above data analysis method (Table S1). Subsequently, genomic DNA of 12 pairs (11 tumor–normal pairs and one normal–normal pair) was mixed in a proportional gradient (0%, 1%, 2%, 5%, and 10% for tumor DNA). The expected allele frequencies of specific variants were verified by ddPCR method (Table S2). Each ddPCR reaction contains 450 nM of each primer, 250 nM of each probe, and 50 ng of genomic DNA input. Manufacturer's recommended protocol was followed as the PCR program was 95°C 10 min, 40 cycles of 94°C 30 s and 60°C 1 min, and 98°C 10 min, with 2°C/min ramp rate. Finally, 60 standard DNA samples at 25 ng/μl concentration were distributed to four commercial NGS‐based genetic testing companies in China for variant detection and TMB calculation based on targeted panel sequencing.

### 2.3 wesTMB calculation in standard samples

Deep WES were performed on two replicates of diluted standard samples, labeled as groups A and B. Sequencing platform was MGISEQ‐2000 (MGI Tech) with PE150 chemistry. The deduped coverage depth for all samples was above 1000×. Sequencing reads were aligned to genome (hs37d5) by Sentieon BWA.^32^ Somatic SNVs/INDELs were called by Sentieon TNscope and annotated by VEP (ensembl release 93). Mutations with <0.01 allele frequency (AF), >.01 PV (*p*‐value by Fisher's exact test of the number of reads supporting the reference and alternate alleles in the tumor and normal samples), ≤8.49 TLOD (log odds that the variant is present in the tumor sample relative to expected noise), ≤26.07 NLODF (log odds that the variant is not present in the normal sample, not a germline variant, given the AF in the tumor sample), >2.74 SOR (symmetric odds ratio to detect strand bias), ≤ −0.39 or >0 MQRankSumPS (*Z*‐score of Alt vs. Ref read mapping qualities per sample), or mutations in repetitive sequences were filtered out (https://support.sentieon.com/appnotes/out_fields/).

### 2.4 psTMB calculation in standard samples

Targeted sequencing utilizing four different commercial panel‐sequencing products were performed on standard samples. The sizes of ROI of these gene panels were similar, ranging from 1.05 to 1.60 Mbp of coding region (Table S3). psTMB was calculated according to their own bioinformatic pipelines. Fitted TMB values and 90% prediction intervals were derived by pretrained fitting association using TMB reported by these companies.

### 2.5 Data availability

The data that support the findings of this study have been deposited into CNGB Sequence Archive (CNSA)^33^ of China National GeneBank DataBase (CNGBdb)^34^ with accession number CNP0001438.

## 3 RESULTS

### 3.1 Linear model between wesTMB and psTMB is applicable to samples with low tumor content

To verify that psTMB estimated by the targeted panels (Table S3) used in this study is quantitatively representative of the wesTMB, we first used publicly available WES datasets MC3 from TCGA with 6704 samples covering 24 different cancer types. wesTMB was calculated as described in the Materials and Methods. For in silico psTMB estimation, mutations mapped to the intersection regions of the exome and the ROIs of targeted gene panels from four vendors (panels A, B, C, D) were kept. As the ROI of targeted panels are biased toward the most frequently mutated genes in cancer, we also optimized the variant retention and exclusion criteria for synonymous mutations and driver mutations as described in the Materials and Methods in order to achieve a better correlation with wesTMB. As shown in Figure S1A, compared to rule 1 and rule 2 (Table 1), we observed the highest Spearman correlation using the new variant retention criteria (New rule) proposed in this study between wesTMB and psTMB generated from panel A. In addition, we observed similar Spearman correlation for all panels based on the new rule.

**TABLE 1:** Inferred regression parameters between psTMB estimated by panel and wesTMB

		Rule 1	Rule 2	New rule
Synonymous mutation		Remove	Retain	Retain
Driver mutation		Retain	Remove	Remove, customized definition
Panel A	Spearman corr.	0.839	0.627	0.872
	Pearson corr.	0.993	0.869	0.993
	*R* ^2^	0.986	0.756	0.986
	Slope	0.910	0.886	0.699
	Intercept	1.335	2.060	0.613
Panel B	Spearman corr.	0.826	0.616	0.861
	Pearson corr.	0.993	0.870	0.993
	*R* ^2^	0.986	0.756	0.986
	Slope	0.941	0.923	0.726
	Intercept	−1.226	2.135	−0.495
Panel C	Spearman corr.	0.817	0.609	0.852
	Pearson corr.	0.992	0.867	0.993
	*R* ^2^	0.983	0.751	0.986
	Slope	0.890	0.887	0.693
	Intercept	−1.499	2.109	−0.670
Panel D	Spearman corr.	0.802	0.605	0.802
	Pearson corr.	0.991	0.864	0.991
	*R* ^2^	0.981	0.747	0.982
	Slope	0.866	0.854	0.928
	Intercept	−1.753	2.113	−1.171

**FIGURE 1:**
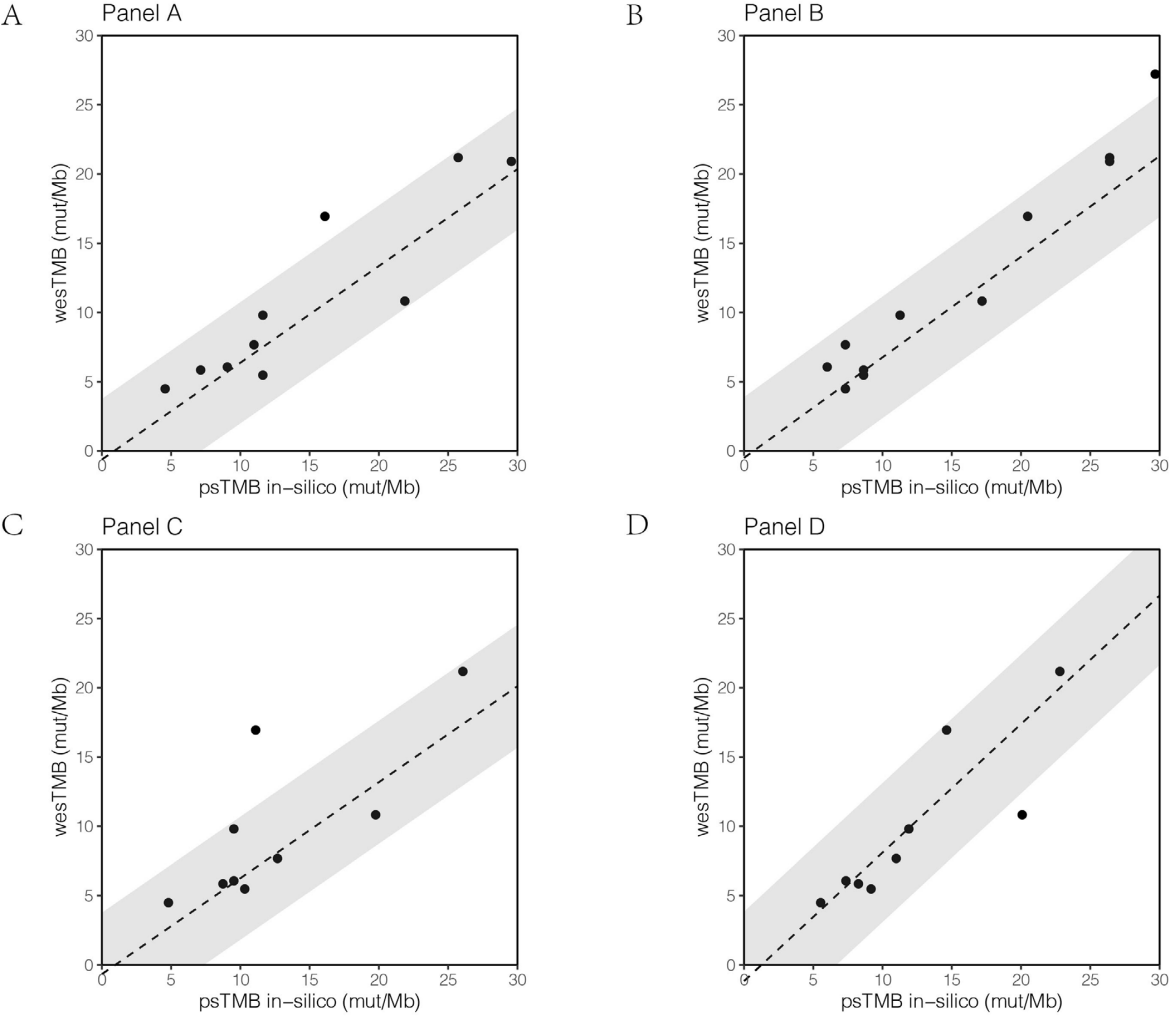
Characterization of raw wesTMB in 11 human tumor cell lines. Raw wesTMB was analyzed by WES of 500× coverage depth. Variants mapped to the shared regions between WES and the ROI of (A) Panel A, (B) Panel B, (C) Panel C, and (D) Panel D were in silico extracted as psTMB. Dashed lines represent fitting formula of the linear model trained by TCGA MC3 datasets, and shadows represent 90% prediction intervals of the linear model

To experimentally validate the quantitative compliance between wesTMB and psTMB, we exploited 11 stable human tumor–normal cell line pairs as shown in Table S1, namely TMB‐1, ‐2, ‐4, ‐5, ‐6, ‐7, ‐8, ‐9, ‐11, ‐12, and ‐13. WES was performed at ∼500× coverage for each cell line pair and wesTMB was calculated ranging from 3.18 to 23.26 mut/Mb (Table S1). Based on the ROI of participating targeted panels, we in silico estimated the psTMB of these tumor cell lines for each panel using our new rule for variant retention (Figure 1). One out of the 11 standard samples mapped out of the 90% prediction interval of linear model developed above for panels A, B, C, and D, respectively (Figure 1). We conclude that psTMB provides a good representation of wesTMB.

**FIGURE 2:**
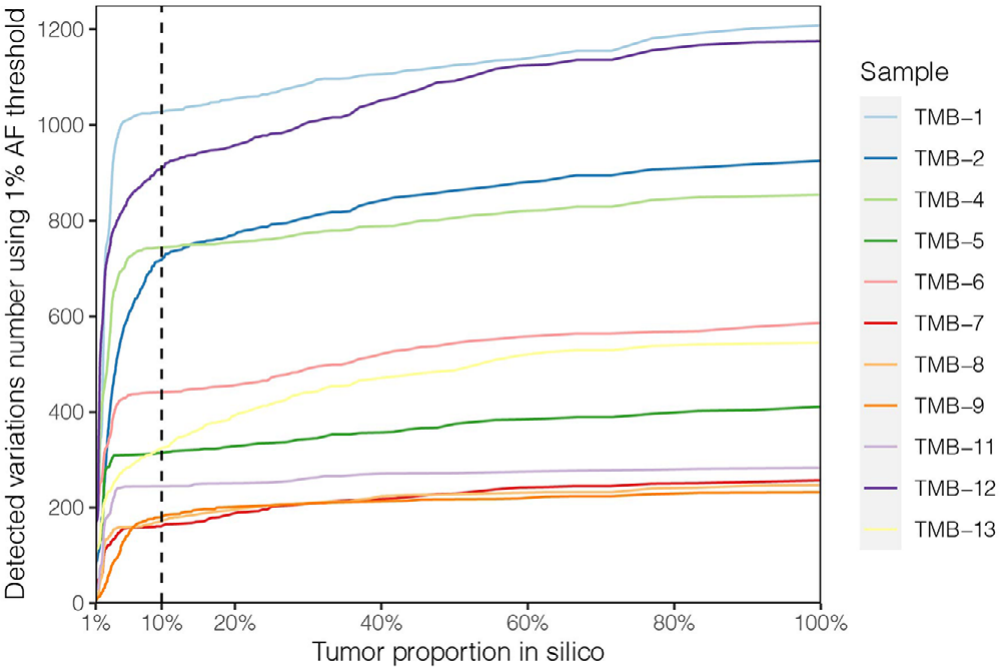
Simulation of the detected number of somatic variants within low tumor content samples. Each of the 11 tumor cell lines (TMB‐1, ‐2, ‐4, ‐5, ‐6, ‐7, ‐8, ‐9, ‐11, ‐12, and ‐13) was in silico simulated individually. Over the gradient of 1% through 100% purity, the AF and the number of supporting reads of each somatic variant are proportionally reduced and checked whether it still satisfies the criteria for variant calling. Variant calling was performed within the diluted sample with 1% AF threshold. Each line reflects an in silico dilution series corresponding to one tumor cell line

However, tumor samples in TCGA datasets normally have a tumor content of more than 20%, the linear model generated from TGGA datasets between wesTMB and psTMB may not be applicable to tumor specimens with low tumor content or liquid biopsy samples considering the different challenges of accurate mutation calling for variants with low allelic frequency (AF) by WES and targeted sequencing methods. We in silico simulated the dilution process of 11 tumor cell lines by proportionally reducing the AF and the number of variant‐supporting reads for each detected somatic variant, and then monitored the total somatic variant count within each sample (Figure 2). The observed variant counts steadily decreased in all the tumor cell lines as the tumor proportion reduced, and then plummeted once the tumor proportion was diluted below 10%, which is dramatically decreased compared to the variant counts of the pure tumor cell lines. We further observed that the decrease rate of observed TMB within diluted samples is dependent on the initial VAF of somatic variants of pure tumor cell lines, and cross‐over of the curves exists at certain tumor content causing shuffling of the TMB value ranking of these samples, which may influence their TMB categorization.

**FIGURE 3:**
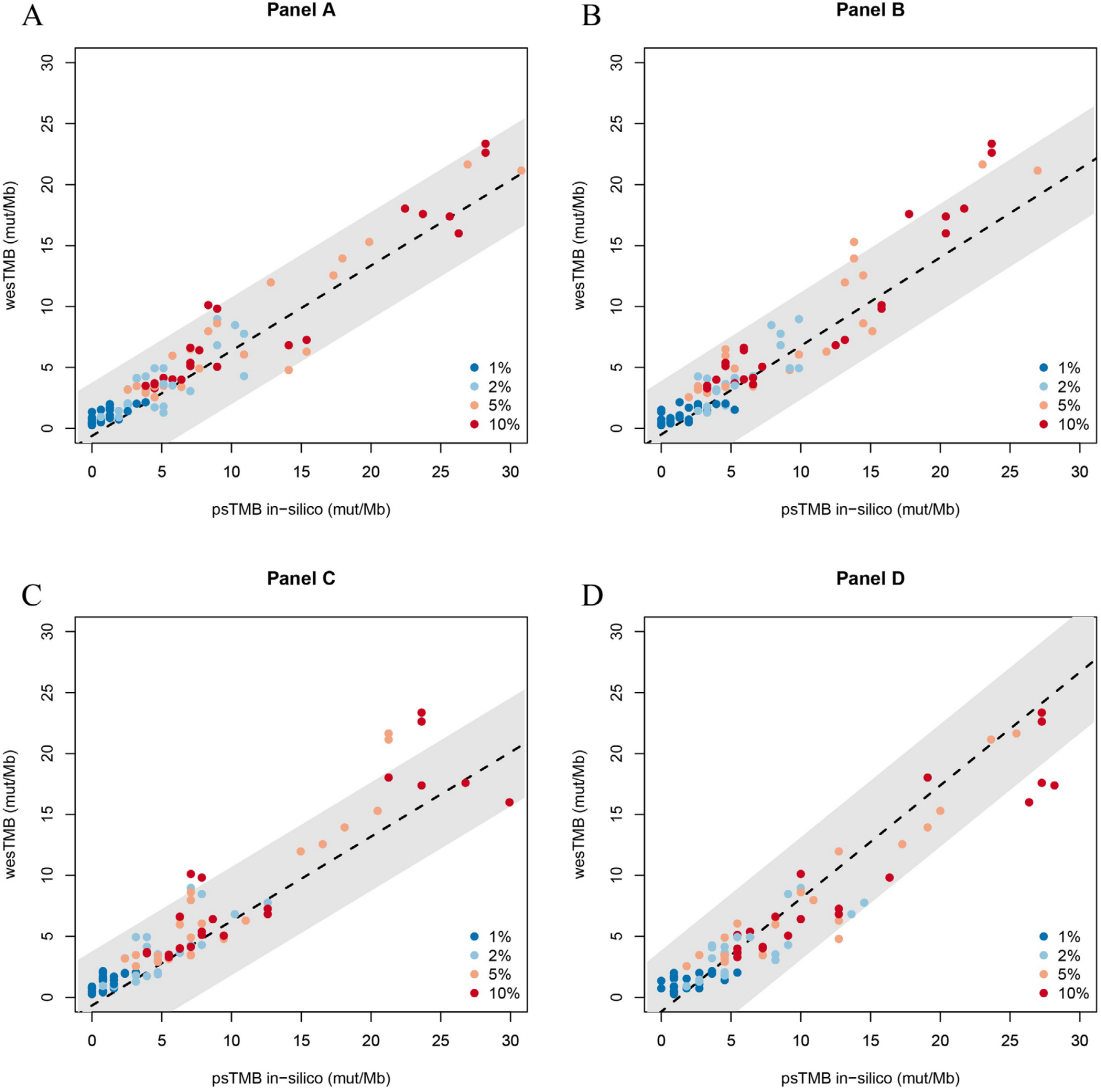
Correlation of observed wesTMB and simulated psTMB in reference standard samples. Variant calling was performed using 1% AF threshold. Eleven sets of low tumor content samples (1%, 2%, 5%, 10%) were analyzed in duplicates, resulting in a total of 88 tests. The numbers of somatic variants mapped to the target ROI of panels A, B, C, and D were individually investigated. Dashed lines represent fitting formula trained by TCGA MC3 datasets, and shadows represent 90% confidence intervals of wesTMB

In order to systematically evaluate the influence of low‐VAF or low tumor content on TMB calculation, we diluted the genomic DNA of these 11 tumor cell lines in those of their matching normal cell lines to gradients of 1%, 2%, 5%, and 10% mass‐to‐mass ratio as our standard samples to mimic samples with various degree of low tumor content. Anticipating the low‐VAF of mutations in these standard samples, we first established their wesTMB using deep WES with an aimed coverage depth above 1000×, a similar depth as targeted panel sequencing in tumor samples, to increase the detection sensitivity of low‐VAF variants. These standard samples were analyzed in replicates as group A and group B. Somatic mutations for wesTMB calculation were analyzed according to the Materials and Methods. An AF threshold for variant calling of 1% was used to compensate the low VAF in corresponding diluted samples. We next extracted the number of somatic variants falling into the targeted ROI of each of the participating vendor's panel as simulated psTMB. The correlation between the observed wesTMB in these diluted standard samples and the simulated psTMB was used to validate the linear model established above with TCGA MC3 dataset. In the two replicates of 11 sets standard samples at four dilution gradients, there were two (2.3%), six (6.8%), seven (8.0%), and four (4.5%) samples falling out of the 90% prediction interval of linear model for panels A, B, C, and D, respectively (Figure 3). These results further supported the validity of linear model between wesTMB and psTMB even in low tumor content samples, although the absolute TMB value may change.

### 3.2 Fitted psTMB is consistent with wesTMB in low‐VAF situations

As accurate TMB quantification is based on reliable mutation detection, we verified the mutation detection performance in diluted standard samples by creating a set of TMB‐14 standards. Unlike the 11 pairs of tumor–normal cell lines, standard sample TMB‐14 was generated by mixing two normal cell lines at 1%, 2%, 5%, and 10% ratio. By controlling the mixing ratio of the two normal cell lines, within which heterozygotic and homozygotic germline variants will display an identical VAF at all germline variant sites unique to each of the samples, we used this ground truth variant set to challenge the variant detection proficiency in WES and each of the participating targeted panels (Figure S1B). Then, we evaluated the mutation detection reproducibility and concordance between the A/B replicates of WES analysis for the 11 pairs of tumor–normal cell lines at gradient dilutions. The mutation detection accuracy was further evaluated by comparing to the ground truth mutations detected in the pure tumor cell lines. Our results showed that reliable variant detection by WES even at above 1000× coverage can only be achieved in the 5% and 10% dilution samples, but not in 1% and 2% dilutions of these cancer cell lines (Figure S1C).

**FIGURE 4:**
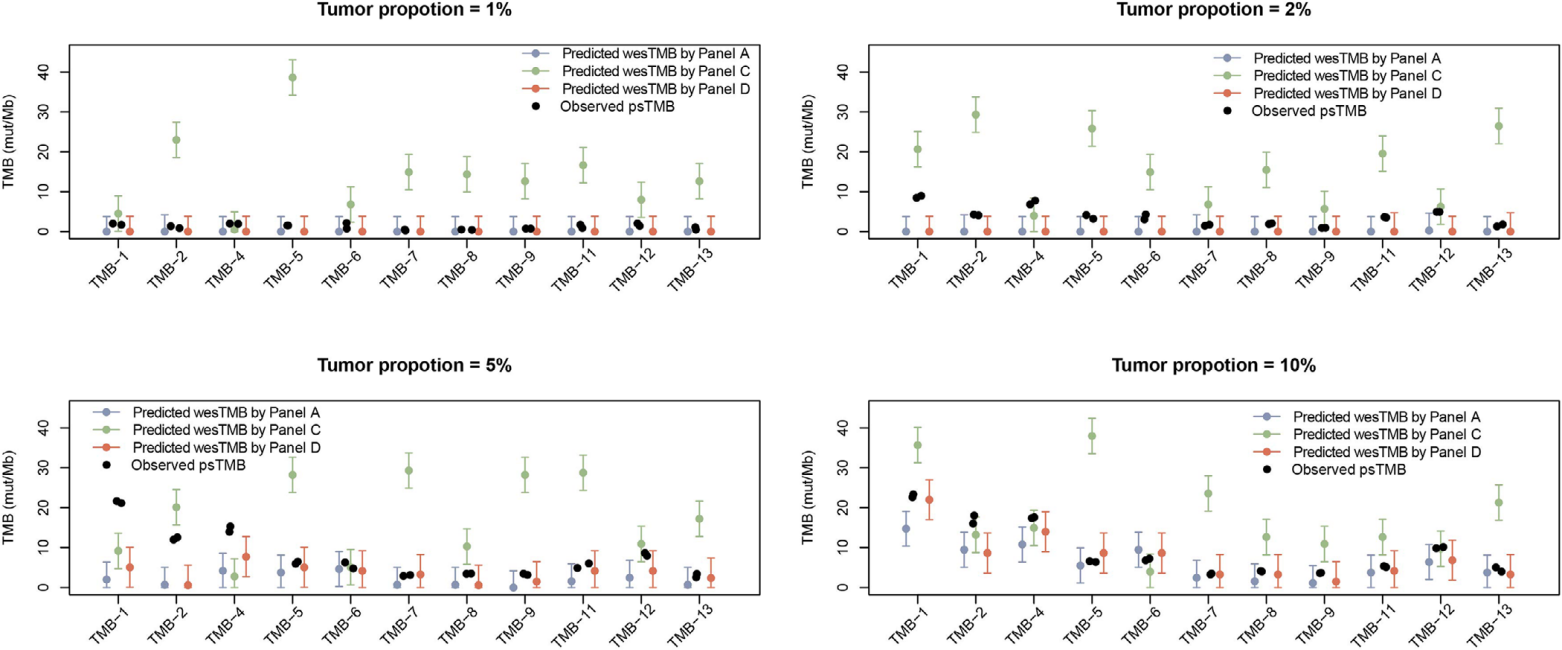
Comparison between observed wesTMB and predicted wesTMB transformed from psTMB by linear model and 90% prediction intervals in reference standard samples with tumor proportions of 1%, 2%, 5%, and 10% (A–D, respectively)

Considering that TMB detection accuracy is likely to be compromised at or below the assay limit of detection (LOD) in low tumor proportion samples, several AF thresholds were experimented when making variant calls (Figure S2). AF threshold was set to 1% for downstream analysis, at which wesTMB was closest to that detected within the undiluted sample. To further evaluate the consistency of TMB estimated by WES and panel sequencing in low tumor content samples, we transformed psTMB through our linear model established above using TCGA dataset to simulated wesTMB. We compared the simulated wesTMB from two vendors with observed wesTMB in these standard samples with different tumor proportions (Figure 4). When tumor proportion was 1% or 2%, neither wesTMB nor simulated wesTMB from psTMB could detect sufficient number of variants to accurately reflect the TMB count in the undiluted samples (Figure S2). When the tumor proportion was 5% or higher, wesTMB detected increased number of variants, with a sensitivity no less than 40% and a positive prediction value no less than 70% (Figure S2). When the tumor proportion was 10%, 8/11 and 10/11 wesTMB fell within the 90% prediction interval of simulated wesTMB derived from the psTMB of panel A and panel D, respectively (Figure 4). Vendor B did not process the standard samples through its psTMB product. When the tumor proportion was 5%, none of panels A, C, and D fell within the 90% prediction interval.

### 3.3 TMB classification performed well in low tumor content samples

Despite of the efforts to establish quantitative agreement between TMB measurements by different experimental methods, it is more accepted that TMB determination should be a qualitative assay by nature for stratifying different patient groups. The clinical relevance of a specific TMB number or a TMB threshold value to distinguish TMB high from low could be achieved only with the support of clinical prognosis information. Considering that our tumor cell line samples are not connected to clinical outcomes, we attempted several sets of previously reported threshold rules for stratifying TMB high or low groups with distinct patient outcomes to determine the TMB status of the 11 tumor cell lines based on their wesTMB value in the pure cell lines, and then evaluate if WES method with compromised variant calling condition for low tumor content samples remains qualified for accurate TMB status determination. We then adopted receiver operative characteristics (ROC) curve to evaluate the results, as this analysis method provides a nonparametric assessment of competency of a binary classifier without predefined threshold for the tested dataset.

**FIGURE 5:**
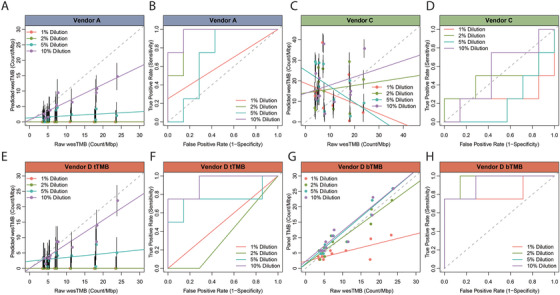
Mutation quantification accuracy and TMB determination proficiency by panels from several vendors in reference standard samples. Each of the 11 standard samples were diluted to 1%, 2%, 5%, and 10% and tested individually. Performance of panel A (A and B), panel C (C and D), panel D with tissue sample TMB pipeline (E and F), and panel D with liquid biopsy sample TMB pipeline (G and H) was separately analyzed. XY scatter plot is presented to show quantitative agreement between the TMB deduced based on the linear model and the wesTMB in undiluted samples (A, C, E, and G). Vertical black bar indicates 90% prediction interval (A, C, and E). ROC curve is presented to show TMB determination proficiency by panels compared to the wesTMB in parental undiluted tumor cell line samples (B, D, F, and H)

A cutoff of 10 mut/Mb is the TMB‐high cutoff approved by FDA and used by many other studies. We first used 10 mut/Mb to distinguish TMB‐1, TMB‐2, TMB‐4, and TMB‐12 as the “high” group from the 11 standards, which produces an equivalent grouping if the top 33% of the standards were considered as high. Using the standard evaluation system, TMB determination by deep WES remains robust in samples with 5% and 10% tumor proportion as expected (Figure S1D, Table S4). TMB count by Vendor D with its TMB pipeline optimized for tissue samples displayed highest quantitative agreement with the anticipated values as well as good potential for TMB status determination. Within the 10% dilution, the 90% prediction interval overlapped with the diagonal trendline. The performance of Vendor D's panel is further strengthened by switching to a bTMB pipeline optimized for liquid biopsy samples (Figure 5G,H). This is expected, as the VAF of somatic variants are often much lower in a cfDNA sample than in a FFPE tissue block. A bTMB pipeline is therefore optimized for the accurate detection of low‐frequency variants. Regrettably, because of the vastly different lower LOD of bTMB pipeline and WES analysis pipeline, it is not possible to construct a linear model for bTMB based on the TCGA dataset. TMB testing from Vendor A underestimated TMB count but showed satisfactory TMB determination potential (Figure 5A,B). It is obvious that panel NGS from Vendor C requires significant optimization, particularly assay specificity, as indicated by the constantly high TMB count in every standard sample and the diagonal ROC curves in all dilution gradients (Figure 5C,D). The correlation coefficient and AUC of each ROC curve is presented in Table S5. Vendor B did not report back the psTMB of the standard samples.

## 4 DISCUSSION

TMB displays its advantage over PD‐L1 IHC staining by providing additional information, such as genetic mutations, which directly educates the pathogenesis of the tumor. However, clinical laboratories offering TMB characterization implement different sequencing platforms, target enrichment panels, experimental methodologies, and bioinformatics analysis pipelines. Lacking a standardized protocol, TMB obtained from different laboratories are poorly translated, sometimes even contradictory, creating dilemmas for oncologists. The TMB Harmonization Project organized by Friends and QuIP provided the basic understanding about TMB measurement, and more investigations promoting precise TMB evaluation are also expected.

Compared to TMB Harmonization Project, this work put a significant emphasis on establishing an evaluation system for samples displaying low cancer cell fraction. Although the number of somatic variants within a sample is constant, the observed TMB count varies depending on VAF of these variants, tumor proportion, and the detection competence of the assay. We value addressing these issues a necessity for a comprehensive evaluation system, as well as furthering the clinical relevance of TMB testing.

In this work, we developed a standardization approach for TMB quantification using standard samples and determined the TMB by WES as reference. First, we constructed linear models to correlate the quantitative agreement between wesTMB and psTMB using TCGA public data. Under the variant retention criteria presented in this study, all four participating panels achieved >0.99 Pearson correlation coefficient in the regression model, indicating that panels targeting as small as 1 Mbp of the coding region of human genome are capable of accurately representing the number of variants in the exome. It is not surprising to find that the slopes of the regression models were all lower than 1, consistent with the fact that panel NGS target regions were biased toward mutational hotspots and the removal of driver mutations from the TMB count is effective. We further investigated how TMB change in response to decreasing tumor proportion through an in silico simulation study using the WES results.

Second, the competence of TMB testing in low tumor content samples was validated through a series of standard samples. Using WES, we analyzed the pattern of false‐positive and false‐negative variant calls within standard sample TMB‐14, as well as the variant detection proficiency in diluted standard samples by comparing to the undiluted samples. We conclude that the standard samples with 1% and 2% tumor proportions are below the assay LOD of wesTMB. Because AF threshold is the key factor to influence trade‐off between tumor proportion LOD and TMB detection accuracy, we also observed that by changing AF threshold to 3%, psTMB fitted best with wesTMB (Figure S3). These multicenter results proved highly consistent between wesTMB and psTMB estimates.

Third, published clinical studies as well as approved indications for TMB testing aim to establish correct TMB high and low grouping, rather than the exact TMB count, as the biomarker predictive to the prognosis of ICB treatment.^1^, ^2^, ^3^, ^4^, ^5^, ^6^ For this reason, we consider TMB testing as a qualitative assay instead of a quantitative assay. We further argue that the ability of the assay to discriminate samples of high TMB from low TMB out‐values the assay's capability at accurately quantifying TMB count within a sample. Following this logic, we chose ROC curve analysis to evaluate the standard samples and our validation strategy, as it functions independently of the quantitative accuracy of the TMB count or a specific threshold value. As a result, panels A and D were qualified while panel C had a poor performance to divide TMB high and TMB low.

Undeniably, this research was limited by following aspects: (1) considering MC3 is a public dataset with relatively low sequencing depth (about 500×), it might not be the optimal estimation for the correlation between our deep sequencing wesTMB and psTMB. (2) Preparing the standard samples at 10% or lower tumor proportion only partially captures the features of TMB testing in liquid biopsy. In addition to lower AF, TMB pipelines dedicated for liquid biopsy need to establish mechanisms to remove the variants originated from clonal hematopoiesis. (3) This set of standard samples consists of only 11 cell line pairs and has no associated prognosis information. The sample size is insufficient to draw any clinically significant conclusion. This set of reference material is only intended for technical validation of the assay system.

In conclusion, this work standardized TMB measurement using low VAF input. Comprehensive evaluations revealed the possible translational value of various TMB panels in practice for impure tumors and liquid biopsy. These standards lay the foundation for precise TMB evaluation, facilitating potential diagnostic and prognostic applications of TMB in clinics. In the future, more exploration around TMB measurement, such as tumor‐only sequencing,^35^ is expected. Investigations in cell line level warranted further clinical substantiation. And clinical trials defining TMB threshold values for ICB therapy response are in demand.

## CONFLICT OF INTEREST

Huan Fang, Dan Li, Tao Liu, Zhenxi Chen, Ke Wang, Shiguang Hao, Zicheng Yu, and Ling Yang are the employees of Geneplus‐Beijing. Xiangyuan Ma, Zhili Chang, and Chenglong Na are the employees of Nanjing Geneseeq Technology Inc. Yin Wang and Jian Bai are the employees of Berry Oncology Corporation. Yanyan Zhang and Fang Chen are the employees of BGI‐Shenzhen. Miao Li and Chao Chen are the employees of YuceBio Technology Corporation. Liangshen Wei and Jinghua Li are the employees of GeneWell Biotech Ltd. The remaining authors declare no conflict of interest.

## AUTHOR CONTRIBUTIONS

*Coordinated the study*: Xin Yi and Jie Huang. *Materials preparation*: Liangshen Wei and Jinghua Li. *Experiments*: Wenxin Zhang, Ruixia Wang, Ling Yang, Dan Li, Yin Wang, Xiaoyan Chang, and Shoufang Qu. *TMB generation*: Yanyan Zhang, Fang Chen, Miao Li, Chao Chen, Jian Bai, Zhili Chang, and Chenglong Na. *Data analysis*: Huan Fang, Tao Liu, Zhenxi Chen, Ke Wang, Shiguang Hao, Xiaoyan Chang, and Xiangyuan Ma. *Manuscript revision*: Zicheng Yu.

## Supporting information



Supporting Information

## Data Availability

The data that support the findings of this study have been deposited into CNGB Sequence Archive (CNSA) of China National GeneBank DataBase (CNGBdb) with accession number CNP0001438.
