# Supplementary material for: Influence of low tumor content on tumor mutational burden estimation by whole‐exome sequencing and targeted panel sequencing
Source: Clin Transl Med. 2021 May 6;11(5):e415. doi: 10.1002/ctm2.415 (PMC8102856; doi:10.1002/ctm2.415)
Supplement: Supplementary file 1 — Supporting Information [file CTM2-11-e415-s001.docx]

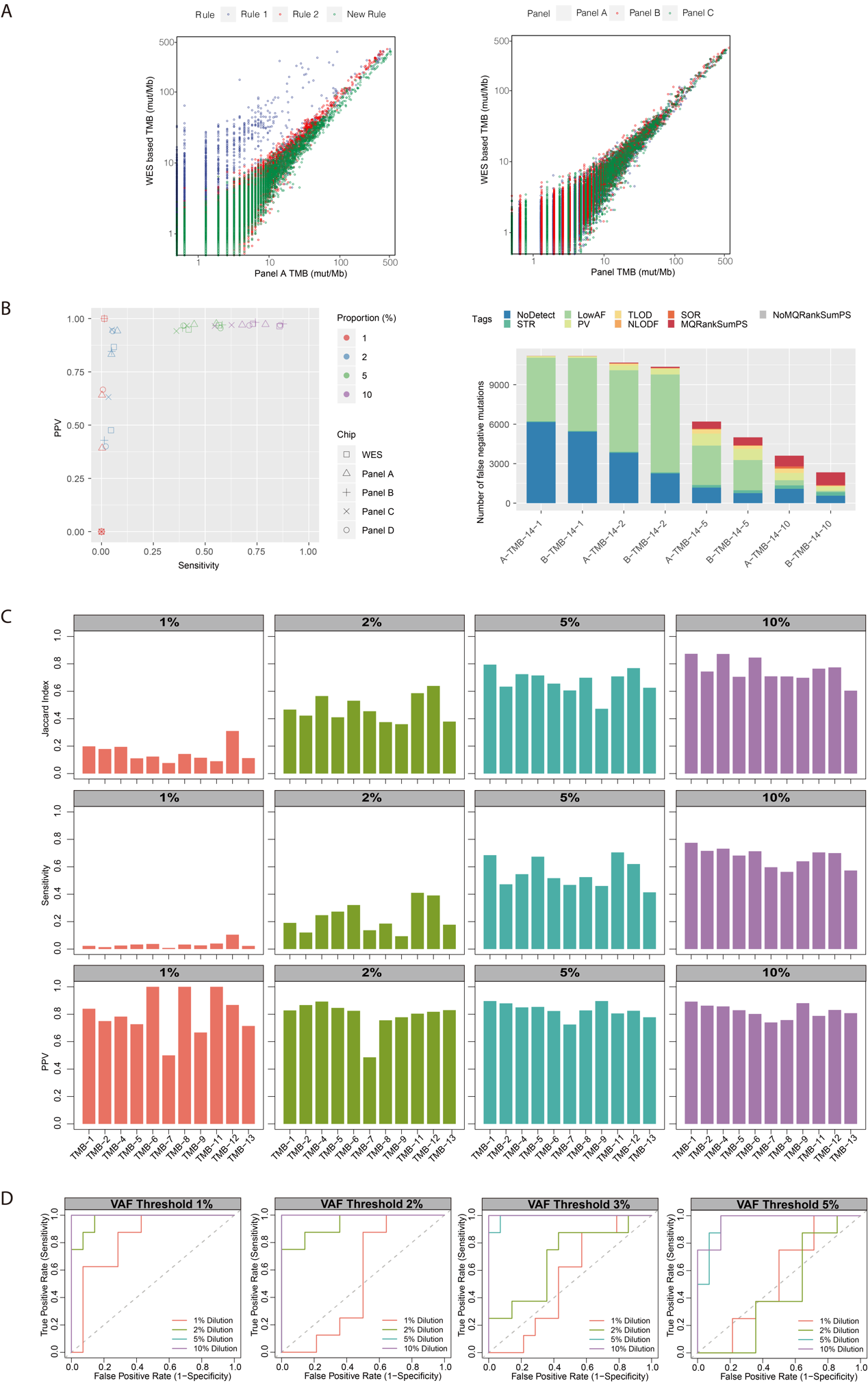


Figure S1. (A) Simulated correlation of psTMB and wesTMB in TCGA MC3 dataset. Scatter plot of wesTMB and several psTMB calculated by different mutation retaining rules using MC3 datasets. (B) Mutation detection performance (PPV and sensitivity) in TMB14 and filter tags statistics for false negative mutations in TMB-14. NoDetect refers to mutations not detected in this sample. LowAF refers to mutations filtered by AF>0.01. NoMQRankSumPS refers to mutations without MQRankSumPS feature. Other tags (STR, PV, TLOD, NLODF, SOR and MQRankSumPS) means mutations filtered by corresponding rules. (C) WES demonstrates competent variant detection proficiency at high VAF. A dilution series of each standard sample was produced at 1%, 2%, 5%, and 10% mass-by-mass ratio with the respective matched normal control. Each step of the gradient was sequenced in duplicates. Consensus variant calls between the two replicates were used for sensitivity and PPV analysis. (D) TMB determination proficiency by WES in diluted standard samples under different bioinformatics parameters. Each of the 11 standard samples were diluted to 1%, 2%, 5%, and 10%, respectively. A bioinformatics AF threshold of 0.01, 0.02, 0.03, and 0.05 were experimented for variant calling. ROC curve is presented to show TMB determination proficiency in the diluted samples compared to the undiluted samples.

Figure S2. wesTMB estimation at a gradient of AF threshold (1-5%) for variant calling. Colors distinguish different tumor proportion. Solid and hollow circles represent two replicates, A and B, respectively. Black squares represent raw wesTMB in undiluted tumor cell lines.

Figure S3. Comparison of wesTMB of different AF thresholds and psTMB approximations derived by different laboratories when tumor proportion is 5% (A) or 10% (B). Samples are sorted by raw_TMB. Line labels 1%, 3% and 5% TMB mean AF threshold is 1%, 3% and 5%. Label fitted_panelA and fitted_panelB mean fitted psTMB derived from vendor A and D.

Table S1. Human cell lines used in this study.

| Pair No. | Cell line ID | White blood cell line ID | Cancer type | Raw TMB (Mut/Mb) |
| --- | --- | --- | --- | --- |
| 1 | TMB-1-H | TMB-1-BL | Adenocarcinoma | 23.56 |
| 2 | TMB-2-H | TMB-2-BL | Adenocarcinoma (non-small cell lung cancer) | 17.79 |
| 3 | TMB-4-H | TMB-4-BL | Adenocarcinoma (non-small cell lung cancer) | 17.97 |
| 4 | TMB-5-H | TMB-5-BL | Small cell lung cancer | 6.97 |
| 5 | TMB-6-H | TMB-6-BL | Small cell lung cancer | 7.38 |
| 6 | TMB-7-H | TMB-7-BL | Primary ductal carcinoma | 3.65 |
| 7 | TMB-8-H | TMB-8-BL | Small cell lung cancer | 4.68 |
| 8 | TMB-9-H | TMB-9-BL | Ductal carcinoma | 3.94 |
| 9 | TMB-11-H | TMB-11-BL | Melanoma | 5.21 |
| 10 | TMB-12-H | TMB-12-BL | Primary ductal carcinoma | 11.21 |
| 11 | TMB-13-H | TMB-13-BL | Primary ductal carcinoma | 5.09 |
| 12 | TMB-14-H (extracted from healthy donor 1) | TMB-14-BL (extracted from healthy donor 2) | NA | - |

Table S2. ddPCR verification of mixing proportions at selected variant sites in the standard samples.

| Mutation | Cell line ID | Raw AF by WES | Mix proportion | Expect AF | Detected AF |
| --- | --- | --- | --- | --- | --- |
| KRAS G12A | TMB-1-H | 100% | 0% | 0% | 0% |
|  | TMB-1-BL | 0% | 100% |  |  |
|  | TMB-1-H | 100% | 1% | 1% | 0.83% |
|  | TMB-1-BL | 0% | 99% |  |  |
|  | TMB-1-H | 100% | 2% | 2% | 1.74% |
|  | TMB-1-BL | 0% | 98% |  |  |
|  | TMB-1-H | 100% | 5% | 5% | 4.29% |
|  | TMB-1-BL | 0% | 95% |  |  |
|  | TMB-1-H | 100% | 10% | 10% | 7.67% |
|  | TMB-1-BL | 0% | 90% |  |  |
| NRAS Q61K | TMB-2-H | 50% | 0% | 0% | 0% |
|  | TMB-2-BL | 0% | 100% |  |  |
|  | TMB-2-H | 50% | 1% | 0.5% | 0.5% |
|  | TMB-2-BL | 0% | 99% |  |  |
|  | TMB-2-H | 50% | 2% | 1% | 1.14% |
|  | TMB-2-BL | 0% | 98% |  |  |
|  | TMB-2-H | 50% | 5% | 2.5% | 2.55% |
|  | TMB-2-BL | 0% | 95% |  |  |
|  | TMB-2-H | 50% | 10% | 5% | 4.78% |
|  | TMB-2-BL | 0% | 90% |  |  |
| TP53 E62* | TMB-4-H | 100% | 0% | 0% | 0% |
|  | TMB-4-BL | 0% | 100% |  |  |
|  | TMB-4-H | 100% | 1% | 1% | 0.89% |
|  | TMB-4-BL | 0% | 99% |  |  |
|  | TMB-4-H | 100% | 2% | 2% | 1.94% |
|  | TMB-4-BL | 0% | 98% |  |  |
|  | TMB-4-H | 100% | 5% | 5% | 4.93% |
|  | TMB-4-BL | 0% | 95% |  |  |
|  | TMB-4-H | 100% | 10% | 10% | 9.36% |
|  | TMB-4-BL | 0% | 90% |  |  |
| SMARCC1 | TMB-5-H | 100% ^a^ | 0% | 0% | 0% |
|  | TMB-5-BL | 0% | 100% |  |  |
|  | TMB-5-H | 100% ^a^ | 1% | 0.5% | 0.61% |
|  | TMB-5-BL | 0% | 99% |  |  |
|  | TMB-5-H | 100% ^a^ | 2% | 1% | 1.17% |
|  | TMB-5-BL | 0% | 98% |  |  |
|  | TMB-5-H | 100% ^a^ | 5% | 2.5% | 2.78% |
|  | TMB-5-BL | 0% | 95% |  |  |
|  | TMB-5-H | 100% ^a^ | 10% | 5% | 5.29% |
|  | TMB-5-BL | 0% | 90% |  |  |
| ARID4A C8T | TMB-6-H | 100% | 0% | 0% | 0.06% |
|  | TMB-6-BL | 0% | 100% |  |  |
|  | TMB-6-H | 100% | 1% | 1% | 1.16% |
|  | TMB-6-BL | 0% | 99% |  |  |
|  | TMB-6-H | 100% | 2% | 2% | 2.7% |
|  | TMB-6-BL | 0% | 98% |  |  |
|  | TMB-6-H | 100% | 5% | 5% | 5.9% |
|  | TMB-6-BL | 0% | 95% |  |  |
|  | TMB-6-H | 100% | 10% | 10% | 11.3% |
|  | TMB-6-BL | 0% | 90% |  |  |
| AMPD2 R843H | TMB-7-H | 100% ^a^ | 0% | 0% | 0% |
|  | TMB-7-BL | 0% | 100% |  |  |
|  | TMB-7-H | 100% ^a^ | 1% | 0.5% | 0.35% |
|  | TMB-7-BL | 0% | 99% |  |  |
|  | TMB-7-H | 100% ^a^ | 2% | 1% | 1.01% |
|  | TMB-7-BL | 0% | 98% |  |  |
|  | TMB-7-H | 100% ^a^ | 5% | 2.5% | 2.29% |
|  | TMB-7-BL | 0% | 95% |  |  |
|  | TMB-7-H | 100% ^a^ | 10% | 5% | 4.04% |
|  | TMB-7-BL | 0% | 90% |  |  |
| TMUB2 N162S | TMB-8-H | 100% | 0% | 0% | 0% |
|  | TMB-8-BL | 0% | 100% |  |  |
|  | TMB-8-H | 100% | 1% | 1% | 0.87% |
|  | TMB-8-BL | 0% | 99% |  |  |
|  | TMB-8-H | 100% | 2% | 2% | 2.05% |
|  | TMB-8-BL | 0% | 98% |  |  |
|  | TMB-8-H | 100% | 5% | 5% | 4.9% |
|  | TMB-8-BL | 0% | 95% |  |  |
|  | TMB-8-H | 100% | 10% | 10% | 9.32% |
|  | TMB-8-BL | 0% | 90% |  |  |
| TP53 Y163C | TMB-9-H | 100% | 0% | 0% | 0% |
|  | TMB-9-BL | 0% | 100% |  |  |
|  | TMB-9-H | 100% | 1% | 1% | 0.85% |
|  | TMB-9-BL | 0% | 99% |  |  |
|  | TMB-9-H | 100% | 2% | 2% | 1.77% |
|  | TMB-9-BL | 0% | 98% |  |  |
|  | TMB-9-H | 100% | 5% | 5% | 4.75% |
|  | TMB-9-BL | 0% | 95% |  |  |
|  | TMB-9-H | 100% | 10% | 10% | 8.4% |
|  | TMB-9-BL | 0% | 90% |  |  |
| BRAF V600E | TMB-11-H | 100% | 0% | 0% | 0% |
|  | TMB-11-BL | 0% | 100% |  |  |
|  | TMB-11-H | 100% | 1% | 1% | 1.3% |
|  | TMB-11-BL | 0% | 99% |  |  |
|  | TMB-11-H | 100% | 2% | 2% | 2.7% |
|  | TMB-11-BL | 0% | 98% |  |  |
|  | TMB-11-H | 100% | 5% | 5% | 6.24% |
|  | TMB-11-BL | 0% | 95% |  |  |
|  | TMB-11-H | 100% | 10% | 10% | 11.1% |
|  | TMB-11-BL | 0% | 90% |  |  |
| TP53 R175H | TMB-12-H | 100% | 0% | 0% | 0% |
|  | TMB-12-BL | 0% | 100% |  |  |
|  | TMB-12-H | 100% | 1% | 1% | 0.97% |
|  | TMB-12-BL | 0% | 99% |  |  |
|  | TMB-12-H | 100% | 2% | 2% | 1.74% |
|  | TMB-12-BL | 0% | 98% |  |  |
|  | TMB-12-H | 100% | 5% | 5% | 4.55% |
|  | TMB-12-BL | 0% | 95% |  |  |
|  | TMB-12-H | 100% | 10% | 10% | 8.91% |
|  | TMB-12-BL | 0% | 90% |  |  |
| TP53 R306 | TMB-13-H | 100% | 0% | 0% | 0.05% |
|  | TMB-13-BL | 0% | 100% |  |  |
|  | TMB-13-H | 100% | 1% | 1% | 0.71% |
|  | TMB-13-BL | 0% | 99% |  |  |
|  | TMB-13-H | 100% | 2% | 2% | 1.28% |
|  | TMB-13-BL | 0% | 98% |  |  |
|  | TMB-13-H | 100% | 5% | 5% | 3.34% |
|  | TMB-13-BL | 0% | 95% |  |  |
|  | TMB-13-H | 100% | 10% | 10% | 6.66% |
|  | TMB-13-BL | 0% | 90% |  |  |

^a^: The cell line is haploid at the variant site.

Table S3. Technical specifications of the participating panel sequencing. Coding region is defined as the exonic regions plus the flanking 2bp both upstream and downstream.

|  | **Number of covered genes** | **Panel size (Mb)** | **Coding region size (Mb)** |
| --- | --- | --- | --- |
| Panel A | 972 | 3.06 | 1.60 |
| Panel B | 783 | 1.65 | 1.22 |
| Panel C | 1622 | 1.58 | 1.50 |
| Panel D | 1326 | 1.55 | 1.05 |

Table S4. Variant detection and TMB status determination in the diluted standard as characterized by WES. Several VAF threshold values were tested at the variant calling step. Variant detection is presented as Pearson correlation coefficient between the WES TMB (Mut/Mb) in the diluted and undiluted sample. TMB status determination is characterized as AUC of ROC curve analysis.

| **VAF Threshold** | **1% Dilution** | |  | **2% Dilution** | |  | | **5% Dilution** | | |  | | **10% Dilution** | | |  |
| --- | --- | --- | --- | --- | --- | --- | --- | --- | --- | --- | --- | --- | --- | --- | --- | --- |
|  | **Corr.** | **AUC** |  | **Corr.** | **AUC** | |  | | **Corr.** | **AUC** | |  | | **Corr.** | **AUC** | |
| 0.01 | 0.610 | 0.830 |  | 0.900 | 0.973 | |  | | 0.978 | 1.000 | |  | | 0.997 | 1.000 | |
| 0.02 | -0.146 | 0.536 |  | 0.660 | 0.938 | |  | | 0.949 | 1.000 | |  | | 0.988 | 1.000 | |
| 0.03 | -0.155 | 0.536 |  | 0.206 | 0.688 | |  | | 0.888 | 0.991 | |  | | 0.974 | 1.000 | |
| 0.04 | -0.162 | 0.518 |  | -0.090 | 0.571 | |  | | 0.866 | 1.000 | |  | | 0.951 | 0.991 | |
| 0.05 | -0.154 | 0.536 |  | -0.259 | 0.438 | |  | | 0.725 | 0.955 | |  | | 0.898 | 0.964 | |

Table S5. Variant detection and TMB status determination in the diluted standard as characterized by panel NGS. Several vendors and bioinformatics analysis pipelines were tested. Variant detection is presented as Pearson correlation coefficient between the wesTMB (Mut/Mb) in the undiluted sample and inferred wesTMB by psTMB. TMB status determination is characterized as AUC of ROC curve analysis.

| **Panel** | **1% Dilution** | |  | **2% Dilution** | |  | **5% Dilution** | | **10% Dilution** | |
| --- | --- | --- | --- | --- | --- | --- | --- | --- | --- | --- |
|  | **Corr.** | **AUC** |  | **Corr.** | **AUC** |  | **Corr.** | **AUC** | **Corr.** | **AUC** |
| Vendor A | 0.000 | 1.000 |  | 0.069 | 0.929 |  | 0.282 | 0.714 | 0.914 | 0.964 |
| Vendor C | -0.330 | 0.250 |  | 0.137 | 0.464 |  | -0.529 | 0.214 | 0.255 | 0.536 |
| Vendor D tTMB | 0.000 | 1.000 |  | 0.000 | 0.714 |  | 0.390 | 0.750 | 0.903 | 0.929 |
| Vendor D bTMB | - | 0.821 |  | - | 0.964 |  | - | 0.929 | - | 0.929 |
